# The Role of Artificial Intelligence in Preservice Science Teachers’ Analogical Reasoning: Evidence from Analogy Design

**DOI:** 10.3390/jintelligence14060110

**Published:** 2026-06-17

**Authors:** Fulya Zorlu

**Affiliations:** Department of Mathematics and Science Education, Ereğli Faculty of Education, Zonguldak Bülent Ecevit University, Zonguldak 67100, Turkey; fulya.zorlu@beun.edu.tr

**Keywords:** analogical reasoning, analogy design, artificial intelligence, preservice science teachers

## Abstract

The study aimed to examine the role of artificial intelligence in preservice science teachers’ analogical reasoning by comparing the features of analogy designs produced with and without artificial intelligence. The research was conducted with 133 preservice science teachers at a public university in Türkiye. Participants were divided into two conditions: those who designed analogies using artificial intelligence (*n* = 62) and those who designed analogies without artificial intelligence (*n* = 71). Analogy design products were analyzed using descriptive analysis, and categorical data derived from these analyses were examined through Pearson’s chi-square tests. In addition, qualitative data obtained from structured interviews with the AI-supported condition were analyzed using content analysis. The results revealed significant differences between the groups in several dimensions of analogy design, presentation format, semantic distance, analogical association, wealth level, and the identification of limitations. Analogies designed with artificial intelligence were more frequently pictorial–verbal, involved both close and remote semantic distance, integrated structural–functional associations, and exhibited extended analogy characteristics. Interview results indicated that preservice science teachers primarily used AI for idea generation, visualization, and creative exploration rather than for generating factual knowledge. These results contribute to the literature by highlighting the potential role of AI in supporting representational transformation processes within science teacher education.

## 1. Introduction

Many fundamental concepts in science education involve abstract structures that cannot be directly observed. Students often attempt to interpret these concepts based on their everyday experiences and intuitive reasoning, which may lead to the development of conceptual structures that do not align with scientific explanations. This situation indicates a mismatch between students’ intuitive knowledge and scientific models (Vosniadou & Brewer, 1992; Chi, 2013; diSessa, 2018). In fact, research suggests that preservice teachers’ scientific explanations vary in quality and that they often require further development to produce higher-level causal explanations (Sakyi-Hagan, 2024). From this perspective, learning scientific concepts cannot be achieved solely through the transmission of correct information; rather, it requires the reorganization and transformation of learners’ existing knowledge structures (Carey, 2000).

According to the constructivist learning perspective, scientific learning is a process of restructuring mental models rather than the direct transmission of knowledge (Driver et al., 2014; Duit & Treagust, 2003). In this process, learners must establish meaningful associations between their existing knowledge structures and target scientific concepts. Learning occurs through the formation of associations among different representations and through transformations between these representations (Duit & Treagust, 2003; Ainsworth, 2006). Within this framework, one of the teacher’s key roles is to transform scientific knowledge into representations that students can understand and to structure appropriate instructional tools that support conceptual transitions (Shulman, 1986; Park & Oliver, 2008).

Among these instructional representations, analogies hold an important place. Analogies function as representational tools that support conceptual understanding by establishing connections between learners’ existing knowledge structures and new scientific concepts (Harrison & Treagust, 1993; Glynn & Takahashi, 1998). They facilitate the construction of mental models by systematically mapping structural–functional associations between a familiar source domain and a target scientific concept (Harrison & Treagust, 1993; Glynn & Takahashi, 1998). However, the effective construction and use of analogies require a cognitive process known as analogical reasoning. Analogical reasoning involves establishing relational mappings between different situations (Vendetti et al., 2014). Ramírez et al. (2024) emphasize that analogical reasoning is not a single-dimensional process but rather a structure consisting of multiple cognitive components. Thus, analogical reasoning is a multidimensional cognitive process that extends beyond surface similarities and involves deeper relational structures.

Consistent with the multidimensional nature of analogical reasoning, various classifications of analogies have been proposed. Gentner (1983) distinguished between surface similarity and structural similarity in analogical reasoning. Curtis and Reigeluth (1984) classified analogies into simple, enriched, and extended forms. In this framework, simple analogies involve brief comparisons based on a single similarity; enriched analogies include multiple similarities but provide limited explanatory detail; and extended analogies present systematic and detailed associations between the source and target domains (Curtis & Reigeluth, 1984). These classifications highlight theoretical frameworks grounded in the cognitive structure and relational depth of analogies.

In addition to their structural features, analogies may vary in their pedagogical presentation and interactional context within instructional processes. Based on Şahin’s (2000, 2003) analyses of classroom use of analogies and examples from the literature (Duit, 1991; Glynn, 1991), analogies can be implemented as simple explanations, story-based analogies, game- or activity-supported analogies, and visual analogies (F. Zorlu & Zorlu, 2022). In this context, simple analogies involve direct comparisons; story-based analogies structure concepts through narrative forms; activity- or game-supported analogies integrate learning into interactive or gamified processes; and visual analogies represent concepts through images, diagrams, or visual models.

Analogies also differ in terms of their instructional functions. Bridging analogies, proposed by Clement (1993), aim to facilitate a gradual transition from students’ alternative conceptions to scientific concepts. Multiple analogies, discussed by Chiu and Lin (2005), contribute to deeper conceptual understanding by explaining the same concept from different perspectives. Accordingly, analogies can be conceptualized as multidimensional representations shaped by relational structure, explanatory scope, level of detail, pedagogical presentation, and functional purpose.

Research has shown that analogies support conceptual understanding (Treagust et al., 1998), enhance explanation generation and reasoning processes (Orgill & Bodner, 2004), and positively influence learning attitudes (Vieira & Morais, 2025). However, when analogical mapping is not appropriately structured, learners may focus on superficial similarities and develop incorrect inferences (Treagust et al., 1998; Glynn, 2007; Orgill & Bodner, 2004). For this reason, the process of constructing analogies is considered not merely an act of information generation but a representational transformation activity that requires cognitive guidance.

In recent years, artificial intelligence (AI) applications have been increasingly examined as tools that provide guidance and adaptive support in learning processes (Zhai et al., 2021). In Türkiye, research on AI in education has also expanded, with recent review-based evidence showing that graduate theses have increasingly addressed the educational uses of AI across different learning contexts (Kaymak et al., 2025). These systems can support learners’ performance through functions such as explanation generation and feedback provision, thereby contributing to instructional decision-making processes (Holmes et al., 2019; Khosravi et al., 2022; Kochmar et al., 2020). Nevertheless, current research primarily focuses on AI’s role in generating instructional materials and lesson plans (Kalenda et al., 2025; Krushinskaia et al., 2024), supporting writing and feedback processes (Kurt & Kurt, 2024; Zhu et al., 2025), or influencing creative reasoning and creative problem-solving performance (Peng et al., 2025; Urban et al., 2024).

However, within the context of teacher education, producing correct explanations alone is insufficient; teachers must also transform scientific knowledge into pedagogical representations that are accessible to learners. Studies examining AI from a teacher education perspective suggest that these technologies may influence not only content production but also instructional design and pedagogical decision-making processes (Zawacki-Richter et al., 2019; Y. Zorlu, 2025). In this sense, AI may support preservice teachers not only in generating content knowledge but also in transforming this knowledge into pedagogical representations that students can understand, thereby contributing to the development of pedagogical content knowledge.

AI is a technology based on modeling cognitive processes specific to human intelligence, such as learning, problem solving, decision-making, perception, and information processing (Russell & Norvig, 2010). In educational processes, AI may contribute to students’ processing, understanding, and structuring of information in accordance with their learning needs (Bulut et al., 2024; Zawacki-Richter et al., 2019). In particular, generative AI tools are associated with students’ information processing and meaning-making processes through functions such as explaining, reorganizing, evaluating, and synthesizing information, guiding the problem-solving process, and providing instant feedback (Kasneci et al., 2023; Wersal et al., 2026). Andersen and Yang (2025) revealed that generative AI has the potential to support higher-order cognitive processes; however, teachers mostly use it for instructional design and administrative efficiency purposes. Nevertheless, the effective and safe use of AI in educational processes depends not only on the opportunities offered by the technological tool but also on designing AI models in alignment with learning objectives, keeping teachers at the center of instructional decision-making processes, and maintaining human oversight (Office of Educational Technology, 2023). Therefore, AI can be considered not as a tool that replaces students’ reasoning processes in education, but as a learning tool that may support cognitive processes through appropriate pedagogical guidance and human oversight.

In this study, AI is used as an external cognitive scaffolding rather than an autonomous source of reasoning. From a distributed cognition perspective, reasoning is not confined to the individual mind but is distributed across learners, tools, representations, and task environments (Ainsworth, 2006; Salomon, 1993; Zawacki-Richter et al., 2019). Accordingly, AI-generated explanations, alternative source domains, prompts, and visual representations may extend preservice teachers’ analogy design processes by providing external resources for comparison, selection, and revision. Recent research also suggests that visual scaffolding in generative AI-supported environments can support learners’ scientific reasoning processes (Lin et al., 2026). This is particularly relevant because analogy design requires source–target mapping, structural–functional association, and awareness of possible mismatches between the source and target domains (Gentner, 1983; Gentner & Colhoun, 2010; Vendetti et al., 2014). From the perspective of cognitive load theory, AI may reduce the extraneous demands associated with generating possible source domains and visualizing abstract astronomy concepts, thereby allowing preservice teachers to allocate more cognitive resources to relational mapping, conceptual accuracy, and the identification of possible mismatches (Plummer & Maynard, 2014; Sneider et al., 2011; Sweller et al., 2019). In addition, external cognitive scaffolding explains how AI may provide temporary support during complex representational tasks without replacing learners’ own reasoning (Kasneci et al., 2023; Office of Educational Technology, 2023). Recent research on generative AI-driven scaffolding also emphasizes that the reliability and quality of AI support depend on reducing hallucinations and maintaining appropriate pedagogical guidance during learning processes (Qian et al., 2026). Therefore, the present study examines whether analogy designs produced in an AI-supported environment differ from those produced without AI in terms of type, presentation, semantic distance, analogical association, state of abstraction, wealth level, and awareness of limitations.

From this perspective, analogy design provides a valuable context for examining AI not merely as a generative tool but as a system capable of offering cognitive guidance during representational transformation processes. Constructing analogies requires learners to establish source–target mappings, identify causal relationships, and recognize potential mismatches (Gentner & Colhoun, 2010). The interactive feedback and relational visualization capabilities of AI suggest that it may support learning during this process. Indeed, limited evidence indicates that AI-based analogy applications can support conceptual structuring (Dai et al., 2024). Although the structural characteristics and pedagogical uses of analogies have been extensively examined in the literature (Glynn, 1991; Harrison & Treagust, 1993; Treagust et al., 1998), empirical studies that comparatively and statistically examine whether AI use creates meaningful differences in preservice teachers’ analogy designs remain limited.

This need becomes particularly evident in topics such as astronomy, where concepts involve extremely large scales and phenomena that are difficult to visualize. In astronomy education, students often struggle to establish causal relationships related to the Earth–Sun–Moon system and the movements of celestial bodies, frequently developing alternative conceptual models (Sneider et al., 2011; Plummer & Maynard, 2014; Cardinot & Fairfield, 2021). Therefore, preservice science teachers’ ability to pedagogically reconstruct these concepts through structured and learner-appropriate analogies can be considered an important indicator of the representation and instructional strategies dimension of pedagogical content knowledge (Magnusson et al., 1999), as well as a reflection of theoretical approaches advocating the deliberate and structured use of analogies in teaching (Glynn, 2007; Treagust et al., 1998). In this context, the study aimed to examine the role of AI in preservice science teachers’ analogical reasoning by comparing the features of analogy designs produced with and without AI. By addressing the role of AI in teacher education not only in terms of content generation but also in relation to representational quality and pedagogical structuring processes, this study seeks to contribute both theoretically and methodologically to the literature.

Accordingly, the study tested the following hypothesis:

**H1.**  *There are significant differences between analogy designs produced by preservice science teachers who use AI and those who do not use AI in terms of one or more analogy features, including type and presentation of analogy, semantic distance, analogical association, state of abstraction, wealth level, limitation, and source domain.*

## 2. Materials and Methods

### 2.1. Research Design

This study was designed as an embedded multiple case study. Case study research aims to investigate phenomena in depth and from multiple perspectives within a specific context (Yin, 2018). In the present study, two different cases were examined: analogy design using AI and analogy design without AI. By comparing these two cases, the study aimed to examine the role of AI in preservice science teachers’ analogical reasoning by comparing the features of analogy designs produced with and without AI.

### 2.2. Participants

The study group consisted of 133 preservice science teachers enrolled in the third year of the Science Teacher Education Program at a public university in Türkiye. The participants took part in analogy design activities within the scope of an astronomy course. The participants were divided into two conditions: the AI-supported condition, also referred to as the AI-supported Analogy Group (AIG), and the control condition, also referred to as the Non-AI Analogy Group (NAIG). The AI-supported condition included 62 preservice science teachers who designed analogies using AI, whereas the control condition included 71 preservice science teachers who designed analogies without using AI. To minimize initial differences between groups, both groups were provided with the same subject content, equivalent time, identical instructions, evaluation criteria, and the same product format (Shadish et al., 2002). This arrangement was implemented as a control strategy to strengthen internal validity.

### 2.3. Prior Knowledge Levels Check

Before the analogy design process, a prior knowledge levels check was conducted with all participating preservice science teachers to examine their prior experience and initial knowledge of analogy design, analogical reasoning and the educational use of AI. In this context, a structured interview form for prior knowledge was used. In the form, the preservice science teachers were asked whether they had previously designed an analogy, what they knew about analogical reasoning and whether they had any previous experience using AI for educational purposes such as analogy design, instructional material development, or science concept representation.

When Table 1 is examined, the responses indicated that none of the preservice science teachers in either group had prior experience in designing analogies. In addition, the most preservice science teachers in both the AIG and the NAIG described analogical reasoning only in general terms, mainly as establishing similarities or relationships between everyday life experiences and scientific subject concepts. Furthermore, none of the preservice science teachers in either group reported prior experience using AI for educational purposes such as analogy design, instructional material development, or the representation of science concepts. Based on these preliminary structured interview responses, the two groups appeared to be comparable in terms of their initial readiness for analogy design and analogical reasoning. However, because this procedure was based on structured self-reported responses rather than a standardized analogical reasoning test, it was used as a baseline readiness check rather than a formal pretest measure.

### 2.4. Data Collection Process

Two data collection methods were used in this study: document analysis and structured interviews. The document data consisted of analogy design products developed by the preservice science teachers. The qualitative data were obtained through a structured interview form including preservice science teachers’ reflections on the analogy design process.

At the beginning of the research process, participants in both the AIG and the NAIG were informed about the purpose, scope, and procedure of the study. Participation was voluntary, and informed consent was obtained from the preservice science teachers. The implementation was carried out face-to-face in a classroom setting within the scope of an astronomy course. Both groups completed the analogy design tasks during scheduled course hours under the researcher’s supervision. Before the analogy design process, astronomy-related topics were taught to all participants over a 10-week instructional period. This instructional period consisted of 20 course hours, corresponding to 1000 min in total. The same astronomy course content, duration, and instructional plan were followed for both groups. The topics were taught by an academician specialized in astronomy education. During these 10 weeks, astronomy-related subjects in the middle school science curriculum were addressed, including the history of astronomy and astronomy topics included in Grades 5–8 science curricula.

Following this instructional period, the preservice science teachers were given four weeks to design their analogies. The analogy design process lasted eight course hours in total, corresponding to 400 min. During the first week, a standard analogy design template was introduced to structure the analogy design process. The template consisted of three sections: “Topic”, “Analogy”, and “Structural Mapping Table”. The preservice science teachers were asked to select an astronomy-related learning objective from the middle school science curriculum, present their analogy in written form, and demonstrate one-to-one and relational correspondences between the source and target domains in the structural mapping table. The preservice science teachers in both groups were allowed to select astronomy-related topics from the same middle school science curriculum framework to preserve the authenticity of analogy-designed tasks.

The weekly implementation process is presented in Table 2.

The researcher provided feedback to both groups during the analogy design process. However, this feedback was limited in scope. The researcher provided feedback only on whether the selected topic was related to astronomy content in the middle school science curriculum and whether the general similarity structure between the everyday, life source domain and the astronomy, related target concept was appropriate. The researcher did not directly generate analogies for participants, did not provide specific source domains, and did not construct source–target mappings on behalf of the participants. The purpose of this feedback was to ensure that the analogy designs were aligned with astronomy topics and that the general analogy structure was understandable, since the preservice science teachers were designing analogies for the first time.

Participants completed the analogy design task individually. They were not allowed to collaborate with peers during the generation, revision, or finalization of their analogies. This procedure was applied consistently in both the control condition, also referred to as the NAIG, and the AI-supported condition, also referred to as the AIG.

The AI-supported condition, also referred to as the AIG, involved preservice science teachers who used AI tools during the analogy design process. The analogy design process was conducted face-to-face in a classroom setting under the researcher’s supervision. The preservice science teachers used their own laptops, tablets, or mobile devices to access AI tools during the process. In the first week, they received theoretical and practical instruction on the concepts of analogy, analogical reasoning, and the use of AI tools in analogy design. The preservice science teachers were allowed to use any AI tools they preferred and were instructed to use AI at all stages of selecting an astronomy-related topic from the middle school science curriculum and analogy design process, including prompt construction, source domain generation, analogy development, visual creation, revision, and refinement. They were then asked to select an astronomy-related topic from the middle school science curriculum and design an analogy appropriate for middle school students (Grades 5–8). The preservice science teachers were not restricted in the type of AI tool they could use. The selection of AI tools, the writing of prompts, the interpretation of AI-generated outputs, and the integration of these outputs into the analogy designs were carried out by the preservice science teachers themselves. During the second week, participants used AI tools to generate possible source domains and develop their initial analogy drafts. During the third week, they evaluated the outputs obtained from AI tools, revised their analogy designs, and checked whether the source–target relationship was scientifically and pedagogically appropriate. The preservice science teachers were explicitly instructed to scientifically verify all output obtained from AI tools. In this process, the scientific appropriateness of the analogies was reviewed by the researcher and an academician (faculty member teaching) the astronomy course. Feedback was provided when elements were identified as scientifically inappropriate, conceptually inaccurate, or pedagogically problematic. During the fourth week, participants finalized and submitted their analogy designs. At the end of the process, the AI-supported condition produced 62 analogy designs. Subsequently, their views on the use of AI in analogy design were collected through a structured interview form.

Although the instructional content, task duration, analogy design template, product format, and researcher feedback were standardized across the two groups, AI tool selection, prompt construction, and interaction patterns were not standardized within the AI-supported group. This decision was made to reflect an ecologically valid AI-supported design environment in which preservice science teachers could use AI tools in ways similar to authentic educational practice. Therefore, the AI-supported condition should be interpreted as a naturalistic AI-supported analogy design environment rather than a tightly controlled AI intervention.

The control condition, also referred to as the NAIG, involved preservice science teachers who designed analogies without using AI tools. The analogy design process was conducted face-to-face in a classroom setting under the researcher’s supervision. The preservice science teachers received theoretical and practical instruction on the concepts of analogy and analogical reasoning but did not use AI tools during the analogy design process. They were instructed to develop their analogies without AI assistance and to rely on their own knowledge, course materials, and traditional learning resources. In the first week, participants were introduced to the concepts of analogy, analogical reasoning, source domain, target domain, and structural mapping. They were then asked to select an astronomy-related topic from the middle school science curriculum and design an analogy appropriate for middle school students (Grades 5–8). During the second week, participants developed possible source domains and prepared their initial analogy drafts without using AI tools. During the third week, they revised their analogy designs based on course knowledge, traditional resources, and researcher feedback. The feedback provided by the researcher was limited to the relevance of the selected astronomy topic and the general appropriateness of the similarity structure between the everyday-life source domain and the astronomy-related target concept. The researcher did not generate analogies, suggest specific source domains, or construct source–target mappings on behalf of the participants. During the fourth week, participants finalized and submitted their analogy designs. At the end of the process, the NAIG produced 71 analogy designs.

The NAIG completed the analogy design task during scheduled classroom hours under the researcher’s supervision. Participants were instructed not to use AI tools during the analogy generation, revision, or finalization process. The researcher monitored the classroom implementation to ensure that participants relied on their own knowledge, course materials, and traditional learning resources.

### 2.5. Data Collection Instruments

Analogy Design Products. A total of 133 analogy design products were analyzed as the primary document data source, including 62 designs from the AIG group and 71 designs from the NAIG group. These products were used to examine structural and qualitative differences between the groups.

Interview Form. A structured interview form consisting of six questions was developed by the researcher to explore preservice science teachers’ experiences with AI use in analogy design. To ensure content validity, the interview form was reviewed by an expert academic in science education. Additionally, the form was piloted with five fourth-year preservice science teachers to evaluate the clarity and comprehensibility of the questions. Necessary revisions were made based on the feedback received. The finalized interview form was distributed to participants through Google Documents, and they were asked to respond to the questions online. Structured interview responses were obtained from all 62 participants in the AIG.

The interview questions addressed topics such as:

- AI tools used during analogy design;
- Purposes of AI use;
- Experiences during the AI-supported analogy design process;
- Types of support provided by AI;
- Strategies used to verify scientific accuracy;
- Advantages of AI-assisted analogy design.

### 2.6. Data Analysis

The data analysis followed the four-stage process proposed by Yıldırım and Şimşek (2016). In the first stage, the data were coded. The data obtained from analogy designs were transferred to Excel 2024 and SPSS 26.0, while the interview responses were transferred to Word and transcribed into written form. To ensure participant confidentiality, preservice science teachers in the AIG group were coded as PST1–PST62. In the second stage, themes and codes were identified. The analogy designs were analyzed using descriptive analysis, whereas the interview data were analyzed using content analysis. In the third stage, themes and codes were reviewed and refined to ensure consistency. At this stage, an independent expert researcher from another university analyzed the data separately. The results were then compared with the researcher’s analysis, and discrepancies were re-examined until consensus was reached. In the final stage, the findings were described and interpreted. The results obtained from the analogy designs were presented using frequency (f) and percentage (%) values, while the interview findings were visualized through concept networks based on frequency values.

The analysis of analogy designs was conducted using descriptive analysis, which involves organizing data according to predetermined categories, presenting findings through frequencies, and interpreting them based on the data (Yıldırım & Şimşek, 2016). The themes and codes used in the analysis were determined based on criteria reported in previous studies (Y. Zorlu & Zorlu, 2021; F. Zorlu & Zorlu, 2022; Hıdır, 2018; Didiş, 2015; Thiele & Treagust, 1994a, 1994b). The analogies were examined under seven themes: “*Type and Presentation of Analogy*”, “*Semantic Distance*”, “*Analogical Association*”, “*State of Abstraction*”, “*Wealth Level*”, “*Limitation*”, and “*Source Domain*”. Under the theme of “*Type and Presentation of Analogy*”, both the presentation format of analogical reasoning and the type of analogy were evaluated. The presentation format was coded as “verbal” or “pictorial–verbal”. The type of analogy was classified as “direct instruction”, “question/discussion”, “story format”, and “gamified”. Under the theme of “*Semantic Distance*”, the semantic distance between the source and target domains was examined. The analogies were coded as “close–remote” and “close”. Under the theme of “*Analogical Association*”, the structure of similarity between the source and target domains was considered. The analogies were coded as “structural”, “functional”, and “structural–functional”. Under the theme of “*State of Abstraction*”, the cognitive state of the source and target domains was examined. The analogies were classified as “concrete–concrete”, “abstract–abstract”, and “concrete–abstract”. Under the theme of “*Wealth Level*”, the richness of the association between the source and target domains was evaluated. The analogies were coded as “simple”, “enriched”, and “extended”. Under the theme of “*Limitation*”, whether the limitations of analogy use were mentioned and whether possible mismatches were indicated by identifying dissimilar aspects of the source and target domains were examined. Both aspects were coded as “exist” and “not exist”. Under the theme of “*Source Domain*”, the feature of the environment in which the analogy was established was evaluated. The analogies were coded as “anthropomorphic”, “environmental”, and “anthropomorphic–environmental” (Hıdır, 2018; Didiş, 2015; Thiele & Treagust, 1994a, 1994b; Y. Zorlu & Zorlu, 2021; F. Zorlu & Zorlu, 2022). Before conducting the Pearson chi-square analyses, the assumptions of categorical data and independence of observations were examined. The variables included in the analyses were categorical, and each analogy design was treated as an independent observation. The Pearson chi-square test was used to examine whether there were statistically significant differences in the distribution of themes and codes between the AIG and the NAIG. When a statistically significant chi-square result was obtained, Bonferroni-corrected post hoc pairwise comparisons of column proportions were conducted to identify which specific codes contributed to the significant group differences. In addition, effect sizes were reported using Cramer’s V. According to Cohen (1988), Cramer’s V values are interpreted as small (0.10), medium (0.30), and large (0.50) effect sizes.

### 2.7. Validity and Reliability

To accurately represent the phenomena examined in this study, the data collection and analysis processes were conducted with careful attention to methodological rigor (Yin, 2018). Continuous interaction with participants throughout the implementation process, along with the combined use of student products (analogy designs) and structured interview data, enhanced data richness and contributed to the validity of the study (Arslan, 2022). Although the AIG was allowed to use them freely, participants were required to critically evaluate and scientifically verify the outputs generated by AI. The NAIG was instructed not to use AI tools during the analogy design process. Both groups completed the analogy design task during scheduled course hours in a face-to-face classroom setting. In both groups, researcher feedback was limited to topic relevance and the general appropriateness of the similarity structure between the everyday-life source domain and the astronomy-related target concept. Therefore, the researcher did not intervene in the actual construction of participants’ analogies, source–target mappings, or final analogy products.

During all stages of the research, consultation was sought from an expert academic from another university. In addition, prior to the implementation, the structured interview form was reviewed by five preservice science teachers enrolled in a science teacher education program at another university to evaluate the clarity and comprehensibility of the questions. These procedures contributed to strengthening the validity and reliability of the study (Creswell & Poth, 2018). In the data analysis process, an expert academic from another university independently conducted content analysis of the qualitative data. To determine inter-coder reliability, the formula (Reliability = Consensus/(Consensus + Disagreement) × 100) proposed by Miles and Huberman (1994) was used. For the analogy design data, the agreement between the two coders was calculated as 95.7%. For the structured interview data, the agreement between the two coders was calculated as 92.8%. According to Miles and Huberman (1994), a reliability value of 80% or higher indicates acceptable reliability in qualitative research. Therefore, the data analysis procedures in this study were considered highly reliable.

## 3. Results

This section presents the findings obtained from the analogy designs and interview data of the preservice science teachers. The results are organized according to the research focus and presented through tables and figures to illustrate the differences between the AIG and the NAIG. Table 3 presents the distribution of the astronomy topics selected by preservice science teachers for their analogy designs.

When Table 3 is examined, it can be seen that preservice science teachers in the NAIG designed analogies on ten different astronomy topics. According to the frequency distribution, the topic of the *Solar System* emerged as the most frequently selected topic. In the AIG, preservice science teachers designed analogies on eight different astronomy topics. Based on the frequency distribution, the *Life Cycle of Stars* and the *Solar System* were the most frequently selected topics.

Table 4 presents the distribution of analogy characteristics according to the themes and codes in both the NAIG and the AIG.

When the “*Type of Analogy*” theme in Table 4 is examined, it can be seen that preservice science teachers in the NAIG predominantly used story format analogies, followed by direct instruction, gamified analogies, and question/discussion-based analogies. In contrast, preservice science teachers in the AIG most frequently used question/discussion-based analogies, followed by story format analogies, direct instruction, and gamified analogies. With regard to the “*Presentation Format of Analogy*” theme, preservice science teachers in the NAIG group mainly presented their analogies in a verbal format, while pictorial–verbal presentations were also frequently observed. In contrast, preservice science teachers in the AIG group predominantly used pictorial–verbal presentations, whereas only 4.8% of the analogies were presented in a purely verbal format. When the “*Semantic Distance*” theme is examined, NAIG participants mostly constructed analogies involving close transfer, while close–remote transfer occurred less frequently. In contrast, preservice science teachers in the AIG group largely constructed analogies involving close–remote transfer, whereas close transfer appeared at a considerably lower rate. In terms of the “*Analogical Association*” theme, NAIG participants primarily used structural associations, followed by structural–functional associations, and functional associations. In contrast, AIG participants predominantly used structural–functional associations, followed by structural associations, and functional associations. Regarding the “*State of Abstraction*” theme, analogies produced by NAIG participants were mostly in the concrete–abstract form, followed by abstract–abstract and concrete–concrete forms. Similarly, in the AIG group, the majority of analogies were also concrete–abstract, while abstract–abstract analogies were observed at a lower rate. When the “*Wealth Level*” theme is considered, NAIG participants mostly produced simple analogies, followed by enriched analogies, and extended analogies. In contrast, AIG participants predominantly produced extended analogies, followed by enriched analogies, while simple analogies appeared considerably less frequently. Within the “*Limitation*” theme, NAIG participants mentioned limitations of analogies and warned about possible mismatches between the source and target domains at relatively lower rates. In contrast, AIG participants more frequently identified limitations in analogy use and warned about potential mismatches between the source and target domains. Finally, when the “*Source Domain*” theme is examined, NAIG participants most frequently used anthropomorphic–environmental domains, followed by environmental domains, and anthropomorphic domains. In the AIG group, analogies were most commonly based on environmental domains, followed by anthropomorphic–environmental domains, and anthropomorphic domains.

To illustrate the characteristics of the analogies designed by preservice science teachers in both groups, one example analogy from each group and its characteristics are presented in Table 5.

When Table 6 is examined, the difference between the groups on the “*Type of Analogy*”, “*Presentation Format of Analogy*”, “*Semantic Distance*”, “*Analogical Association*”, “*Wealth Level*”, “*Limitation*”, “*Warning about possible mismatches*”, and “*Source Domain*” themes were found to be statistically significant, “*Type of Analogy*”: *χ* ^2^(3, N = 133) = 31.13, *p* < .001, Cramer’s V = 0.484, “*Presentation Format of Analogy*”: *χ* ^2^(1, N = 133) = 35.17, *p* < .001, Cramer’s V = 0.514, “*Semantic Distance*”: *χ* ^2^(1, N = 133) = 58.90, *p* < .001, Cramer’s V = 0.665, “*Analogical Association*”: *χ* ^2^(2, N = 133) = 25.71, *p* < .001, Cramer’s V = 0.440, “*Wealth Level*”: *χ* ^2^(2, N = 133) = 43.67, *p* < .001, Cramer’s V = 0.573 “*Limitation*”: *χ* ^2^(1, N = 133) = 35.39, *p* < .001, Cramer’s V = 0.516, “*Warning about possible mismatches*”: *χ* ^2^(1, N = 133) = 12.51, *p* < .001, Cramer’s V = 0.307, “*Source Domain*”: *χ* ^2^(2, N = 133) = 9.24, *p* < .05, Cramer’s V = 0.264. The effect size of “*Presentation Format of Analogy*”, “*Semantic Distance*”, “*Wealth Level*” and “*Limitation*” indicates a large association between the variables. The effect size of “*Type of Analogy*”, “*Analogical Association*” and “*Warning about possible mismatches*” indicates a moderate association between the variables. The effect size of “*Source Domain*” indicates a small association between the variables. No statistically significant difference was found between the groups in the theme “*State of Abstraction*”, *χ* ^2^(2, N = 133) = 2.06, *p* > .05, Cramer’s V = 0.124.

When the codes under the “Type of Analogy”, “Presentation of Analogy”, “Semantic Distance”, “Analogical Association”, “Wealth Level”, “Limitations in the use of analogy”, “Warning About Misleading Matches”, and “Source Domain” themes in Table 7 were examined, statistically significant differences were found between the groups (*p* < .05). The control condition (NAIG) was associated with higher proportions of “direct instruction”, “story format”, “verbal presentation”, “close semantic distance”, “structural association”, “simple analogies”, “not exist of limitations in the use of analogy”, “not exist of warning about misleading matches”, and “anthropomorphic–environmental source domains”. In contrast, the AI-supported condition (AIG) was associated with higher proportions of “question/discussion format”, “pictorial–verbal presentation”, “close–remote semantic distance”, “structural–functional association”, “extended analogies”, “exist of limitations in the use of analogy”, and “exist of warning about misleading matches”.

When Table 8 is examined, the theme “*Purpose of AI Use*” includes eight different codes: “idea generation”, “visual creation/enhancement”, “idea development/refinement”, “text editing/expression improvement”, “information search/research”, “story construction”, “prompt preparation”, and “originality checking”. According to the frequency distribution, the most prominent codes were “idea generation”, “visual creation/enhancement”, and “idea development/refinement”. Some preservice science teachers explained that they used AI to generate and refine ideas during the analogy design process. For example:

− *“I selected the topic*, *asked AI for possible analogies*, *chose the most appropriate ones*, *and then checked their scientific accuracy.”* (PST1)
− *“First we thought about our own analogy*, *and then we sent it to AI and asked it to improve it.”* (PST5)
− *“It was very detailed and helpful during the storytelling stage.”* (PST23)
− *“It helped us develop our initial idea and offered different possibilities.”* (PST34)
− *“It inspired us*, *and thanks to this inspiration we generated new ideas.”* (PST36)
− *“I used ChatGPT to prepare the prompt and then applied that prompt in Gemini.”* (PST45)

When the theme “*Support Provided by AI*” is examined, six codes were identified: “visual support”, “time saving”, “creativity support”, “facilitating the process”, “diversity of ideas”, and “creating a basic framework”. According to the frequency distribution, the most prominent codes were “visual support”, “time saving”, “creativity support”, “facilitating the process”, and “diversity of ideas”. Participants emphasized that AI supported both visual production and the efficiency of the process. For instance:

− *“It provided significant support in creating visuals appropriate for the analogy.”* (PST18)
− *“It saved time and provided quick solutions.”* (PST26)
− *“It accelerated the process and helped me save time.”* (PST51)

Within the theme “*AI Use Process*,” four codes were identified: “selecting from AI-generated examples”, “AI-assisted idea development”, “trial-and-error prompt writing”, and “using multiple AI tools”. According to the frequency distribution, the most prominent codes were “selecting from AI-generated examples” and “AI-assisted idea development”. When the theme “*Using AI and Writing Prompts*” was examined, four codes emerged: “learning to write prompts”, “the importance of clear instructions”, “training AI through interaction”, and “selecting appropriate AI tools”. According to the frequency distribution, the most prominent code was “learning to write prompts”. Participants emphasized that the process helped them improve their ability to interact effectively with AI tools:

− *“I learned how to use artificial intelligence more effectively.”* (PST39)
− *“I learned how to write prompts during this process.”* (PST44)
− *“I realized how important it is to write prompts clearly.”* (PST58)

When the theme “*Scientific Control When Using AI*” was examined, four codes were identified: “the need to verify scientific accuracy”, “the necessity of teacher supervision”, “comparing multiple sources”, and “the risk of generating incorrect information”. According to the frequency distribution, the most prominent code was “the need to verify scientific accuracy”. Participants emphasized that AI-generated outputs needed to be critically evaluated:

− *“Artificial intelligence can sometimes produce scientifically incorrect information*, *so I had to check it.”* (PST12)
− *“I learned that how clearly I ask the question affects the accuracy of the result.”* (PST42)

Within the theme “*Preferred AI Tools*” the codes included “Gemini”, “ChatGPT”, “Canva”, “Google Flow”, and “CapCut (AI-assisted editing)”. According to the frequency distribution, “Gemini” emerged as the most frequently used AI tool. Participants explained that they often used multiple AI tools for different purposes:

− *“We used Gemini to create visuals appropriate for the analogy.”* (PST3)
− *“We used ChatGPT*, *Gemini*, *and Canva together. One generated ideas*, *another created visuals*, *and the other helped design the template.”* (PST30)
− *“I used ChatGPT to simplify astronomy concepts and generate alternative analogy ideas.”* (PST41)

The definitions of the themes and codes presented in Table 8 are provided in Appendix A.

## 4. Discussion

This study examined group-based differences in preservice science teachers’ analogy designs produced with and without AI. The results showed that AI-supported analogy designs were more frequently pictorial–verbal, close–remote in semantic distance, structurally–functionally integrated, extended in wealth level, and more likely to include explicit limitations and warnings about possible misleading matches. These results should be interpreted as differences associated with AI use rather than as direct causal effects of AI.

When the results of the study are evaluated in terms of presentation format, it was found that preservice science teachers who designed analogies using AI predominantly presented their analogies in a pictorial–verbal format, whereas those who designed analogies without AI mainly presented them in a verbal format. According to these results, the use of AI not only facilitated content generation in preservice science teachers’ analogy designs but also contributed to the construction of richer explanatory structures in which visual and verbal elements were used together by transforming the representational form of the analogies. In this context, the ability of generative AI tools to accelerate and facilitate the production of visual content (Kalenda et al., 2025; Zhai et al., 2021) may have enabled preservice science teachers to enrich their analogy designs with visual elements rather than limiting them to textual explanations. The greater use of pictorial–verbal representations in the AIG can be interpreted through distributed cognition and multiple representation theory. AI-generated visual outputs may have served as external representational resources that helped preservice science teachers organize astronomy concepts through both verbal and visual modes. The preservice science teachers’ views directly support this result. Participants stated that they primarily used AI for creating visuals and adapting visual representations to their analogies. This suggests that the use of visual representations in analogy design was not incidental but rather a deliberate pedagogical choice. This result is consistent with multiple representation theory. Ainsworth (2006) emphasizes that learners’ ability to transition between different representational forms clarifies conceptual relationships and strengthens cognitive integration. In subjects such as astronomy, where spatial relationships and causal processes are central, purely verbal representations may be insufficient, whereas visually supported representations facilitate the construction of mental models (Plummer & Maynard, 2014; Sneider et al., 2011). Therefore, the preservice science teachers’ emphasis on visual creation/enhancement indicates that the observed increase in pictorial–verbal presentations is not coincidental but reflects a conscious pedagogical decision. The use of AI appears to be associated with the development of visually supported analogy designs. Through AI-assisted visualization, preservice science teachers may have structured abstract astronomical concepts in ways that are more compatible with students’ mental models. In this context, the use of AI may have facilitated not only visual production but also the deliberate construction of visual-verbal analogies by preservice science teachers to make astronomy concepts more understandable.

One of the most striking results of the study is the large effect size observed in the semantic distance dimension (Cramer’s V = 0.665). The analogies produced by the NAIG largely remained at the level of close semantic distance, whereas the analogies produced by the AIG tended to involve close–remote semantic distance. In the group that did not use AI, the fact that analogies mostly remained at the level of close semantic distance suggests that these designs were largely based on superficial or one-dimensional similarities. In contrast, the prominence of analogies involving close–remote semantic distance in the group that used AI indicates that preservice science teachers were able to establish deeper, relational, and causal connections between the source and target domains. AI may have expanded the range of accessible source domains because of the higher frequency of close–remote semantic distance in AI-supported designs. From a distributed cognition perspective, AI-generated alternatives may have expanded the representational environment in which preservice science teachers compared and selected possible analogical sources. This result is consistent with Gentner’s (1983) structure-mapping theory, as the strength of analogical reasoning depends not on superficial similarities but on the quality of relational mappings (Holyoak, 2012). It is also consistent with cognitive processes in modeling (Baba et al., 2022; Y. Zorlu & Sezek, 2019, 2020; Y. Zorlu & Zorlu, 2020). Similarly, Vendetti et al. (2014) stated that strengthening relational mapping increases the cognitive value of analogies. In this context, the alternative ideas and examples provided by AI may have functioned not only by enabling preservice science teachers to access different analogy options but also as a cognitive stimulus that expanded their relational reasoning and facilitated remote transfer by making the process, cause–and–effect, and functional associations between concepts more visible. Therefore, this result is also consistent with recent studies emphasizing the potential of generative AI systems to diversify idea generation and facilitate access to information in higher education contexts (Abdallah et al., 2025).

Another important result is that structural–functional associations were dominant in the analogies produced by the AIG. The prominence of structural–functional associations in the analogies produced by the group that used AI indicates that the analogy designs did not remain merely at the level of physical similarities; rather, they evolved into a more explanatory structure through process-, function-, and mechanism-based connections. According to this result, the analogies were based not only on physical similarities but also on process-based and functional associations. The prominence of structural–functional associations in the AIG can be discussed in relation to cognitive load theory. By reducing the initial burden of generating source domains and visualizing abstract astronomy phenomena, AI may have allowed preservice science teachers to allocate more attention to relational mapping and causal-process explanations. Harrison and Treagust (1993) emphasize that effective analogies should not rely solely on physical similarities but should also include mappings at the level of processes and mechanisms. From this perspective, it can be concluded that the use of AI may have contributed to the preservice science teachers’ shift from object-to-object similarities toward process-to-process relationships in the analogical mapping process. This contribution is particularly important in explaining astronomy concepts because the understanding of such concepts is strengthened not only through visual or structural similarities but also through functional explanations that make the operation of events and cause–and–effect relationships visible. Therefore, AI can be considered not merely as a tool that provides content diversity in analogy design, but also as a cognitive resource that supports preservice science teachers’ relational mapping and process-based reasoning skills. This inference is also consistent with studies emphasizing the importance of relational mapping and process-level connections in the multidimensional structure of analogical reasoning (Ramírez et al., 2024; Treagust et al., 1998).

Another strong result of the study is the large effect size observed in the wealth level dimension (Cramer’s V = 0.573). According to this result, considering that extended analogies are multilayered structures that include multiple similarities, explanations, comparisons, and causal relationships (Curtis & Reigeluth, 1984), the fact that more than half of the analogies produced by the AIG were classified as extended analogies indicates that analogy design became more multidimensional. The qualitative results support this result, particularly through codes such as creativity support, diversity of ideas, and creating a basic framework. Considering that extended analogies are structures that include multiple mappings, explanations, and comparisons (Curtis & Reigeluth, 1984), the provision of alternative ideas, visual options, and different forms of representation by AI tools may have facilitated preservice science teachers’ transformation of their analogies from a single similarity into richer relational structures. This inference is consistent with studies emphasizing that multilayered relational structures support conceptual change (Duit & Treagust, 2003) and with recent systematic reviews indicating that AI systems can offer multiple alternatives in idea-generation processes (Munaye et al., 2025).

One of the most critical pedagogical dimensions of analogies is the explicit articulation of their limitations (Glynn, 2007). Treagust et al. (1998) argue that analogies may lead to incorrect generalizations if their limitations are not clearly stated. In the present study, the proportion of analogies that explicitly addressed limitations reached 72.6% in the AIG, which is noteworthy. This may be related to the ability of AI systems not only to generate ideas but also to highlight potential weaknesses and mismatches in analogical explanations. Steiss et al. (2024) reported that ChatGPT can provide suggestions for improving student writing, although the quality of feedback may remain limited compared with human evaluators. Similarly, Benčina and Dubac Nemet (2025) found that systems such as ChatGPT and Google Gemini provide feedback not only on content but also on structure, coherence, and flow, although differences exist between models in terms of feedback organization and depth. In this context, the results of the present study suggest that AI tools may serve a supportive role in analogy design, but the quality of feedback should still be filtered through pedagogical judgment and human guidance. This may have helped preservice science teachers identify the limitations of their analogies and explicitly state potential mismatches between the source and target domains. Furthermore, interview results revealed that preservice science teachers were aware that AI systems may produce incorrect information, which led them to verify scientific accuracy and compare multiple sources. This awareness may explain why the participants in the AIG were more likely to include explicit warnings about limitations and potential mismatches in their analogy designs. This result is consistent with recent research highlighting the reliability limitations of AI systems and the need for pedagogical supervision in educational contexts (Holmes et al., 2019; Munaye et al., 2025; Ramírez et al., 2024).

The absence of a significant difference in the state of abstraction dimension indicates that both groups tended to construct concrete–abstract mappings in astronomy topics. This situation is likely related to the inherently abstract nature of astronomy concepts (Cardinot & Fairfield, 2021; Vosniadou & Brewer, 1992). Therefore, regardless of whether preservice science teachers used AI, their use of concrete source domains to make astronomical events more understandable is an expected situation. Therefore, it is expected that preservice science teachers in both groups would use concrete representations to explain abstract astronomical phenomena.

Another important result emerging from the interviews is that preservice science teachers developed awareness of prompt writing during the AI interaction process. Awareness regarding the quality of prompt construction during interaction with AI reveals that preservice science teachers began to consider AI as an interactive learning tool that should be guided according to pedagogical purposes. This finding is consistent with recent evidence indicating that AI-assisted pedagogical tasks, such as question generation, can support preservice science teachers’ 21st-century teaching competencies and instructional design practices (Y. Zorlu, 2025). Zawacki-Richter et al. (2019) note that research on AI in teacher education has largely focused on outputs rather than on the processes of representation and pedagogical design. The present study contributes to this literature by showing that preservice science teachers developed digital pedagogical awareness during the analogy design process. The preservice science teachers emphasized the need to verify scientific accuracy, compare multiple sources, and remain aware of the risk of incorrect AI-generated information. This result is consistent with recent research showing that reliable generative AI-driven scaffolding requires reducing hallucinations and enhancing the quality of support provided to learners (Qian et al., 2026). Therefore, AI-supported analogy design should be understood not as an autonomous production process, but as a pedagogically guided and critically monitored design process.

The preservice science teachers’ more frequent use of Gemini and ChatGPT suggests that the choice of AI tools in the analogy design process may be influenced by practical variables such as accessibility, ease of use, and free access opportunities. One possible reason for the high use of Gemini may be that Google provided university students with free access to the Gemini AI Pro version for one year, making it more accessible than other tools. This situation indicates that preservice science teachers’ choices of digital tools are influenced not only by pedagogical functionality but also by access conditions.

### Limitations of This Study

This study has several limitations that should be considered when interpreting the findings. The study was limited to preservice science teachers enrolled at a public university in Türkiye. The study was limited to using a quasi-experimental grouping structure rather than random assignment; therefore, the findings should be interpreted as group-based differences associated with AI use rather than direct causal effects. Although a prior knowledge check was conducted, no standardized analogical reasoning pre-test was administered. Although both groups selected astronomy topics from the same middle school science curriculum framework, the distribution of selected topics differed between groups. Preservice science teachers in the AIG were allowed to use different AI tools, such as Gemini, ChatGPT, Canva, and other AI-based applications, according to their own preferences and needs during the analogy design process. The term AI-supported analogy design in this study refers to the use of AI as an external cognitive scaffolding tool during the design process. The intervention was limited to a four-week analogy design process following astronomy instruction. Therefore, the study provides evidence regarding analogy designs produced within a short-term instructional context. The study focused on analogy design products and interview responses.

## 5. Conclusions

The study examined the role of AI in preservice science teachers’ analogical reasoning by comparing the features of analogy designs produced with and without AI. AI may function not merely as a content-generation tool, but as an external cognitive scaffold that is associated with representational enrichment, relational structuring, and the identification of possible mismatches during analogy design. Across multiple dimensions of analogy design, significant differences were observed between participants who used AI and those who did not. In particular, analogies produced with AI demonstrated a higher tendency toward relational mapping, close–remote semantic distance, and structural–functional integration, all of which are central components of analogical reasoning. These findings suggest that AI may facilitate a shift from surface-level similarity toward deeper relational processing, aligning with theoretical perspectives that conceptualize analogical reasoning as a core component of higher-order cognition. Furthermore, the increased prevalence of extended analogies and explicit identification of limitations in the AI-supported condition group indicates that AI may support not only the generative aspects of reasoning but also metacognitive awareness. The ability to recognize mismatches and boundaries between source and target domains reflects a more sophisticated level of cognitive processing, suggesting that AI-supported environments may promote reflective reasoning alongside representational construction. Importantly, the absence of a significant difference in abstraction levels across groups indicates that AI does not fundamentally alter the nature of analogical representation (e.g., concrete–abstract mapping) but rather enhances the quality and organization of relational structures within those representations. This distinction highlights the role of AI as a facilitator of cognitive structuring rather than a determinant of representational form. Overall, the findings contribute to the literature on intelligence and cognition by providing empirical evidence that AI can support the development of analogical reasoning through mechanisms such as relational expansion, representational enrichment, and metacognitive regulation. In this sense, AI may be conceptualized as an external cognitive scaffold that extends learners’ capacity to construct, evaluate, and refine complex relational representations.

Future studies examining AI-assisted analogy design are recommended to be conducted with students at different educational levels and across various science topics. In addition, investigating analogy design processes in relation to variables such as conceptual understanding, cognitive load, analogical reasoning, and academic achievement may provide more comprehensive insights. It is also recommended that future research examine the effects of AI use in analogy design through quantitative, qualitative, and mixed-method research designs to provide a multidimensional understanding of the phenomenon. Although the present study focused on analogy design, future studies should also explore the role of AI in other pedagogical processes such as experiment design, activity development, and instructional planning. Finally, research examining the role of prompt quality in determining analogy richness and semantic distance levels, as well as comparative studies investigating the effects of different AI tools on the quality of analogical mapping, may make significant contributions to the literature.

## Figures and Tables

**Table 1:** Preservice science teachers’ prior knowledge levels for analogy design and analogical reasoning.

Preservice Science Teachers’ Prior Knowledge Levels	NAIG		AIG	
	*f*	*%*	*f*	*%*
Had no previous analogy design experience	71	100.0	62	100.0
Defined analogical reasoning only as establishing similarities/relationships between everyday life and scientific concepts	53	74.6	47	75.8
Reported systematic/theoretical knowledge of analogy design	18	25.4	15	24.2
Had no previous experience using AI for educational purposes such as analogy design or instructional material development	71	100.0	62	100.0

**Table 2:** Weekly implementation process of the analogy design.

Week	AIG	NAIG	Duration	Researcher Role
First	Introduction to analogy, analogical reasoning, and AI use in analogy design	Introduction to analogy and analogical reasoning	2 course h/100 min	Introduction of task instructions and the analogy design template
Second	Developing the source domain and initial analogy draft using AI tools	Developing the source domain and initial analogy draft without AI tools	2 course h/100 min	Feedback limited to topic selection
Third	Evaluating AI outputs and revising the analogy design	Revising the analogy design using traditional resources	2 course h/100 min	Feedback on the general similarity structure between the everyday-life source domain and the astronomy-related target concept
Fourth	Finalizing and submitting the analogy designs	Finalizing and submitting the analogy designs	2 course h/100 min	Collection of analogy design products

**Table 3:** Topics of the analogies designed by preservice science teachers.

Topic	NAIG (*f*)	AIG (*f*)
Solar System	36	16
Formation of the Seasons	8	9
Solar and Lunar Eclipses	7	2
Motions and Phases of the Moon	6	6
Life Cycle of Stars	4	16
Structure and Characteristics of the Moon	2	–
Understanding Our Planet Earth	2	–
Galaxies/Types of Galaxies	2	6
Telescopes	2	–
Sun–Earth–Moon System	2	4
Universe	–	3

**Table 4:** Findings related to the characteristics of the analogies.

Theme	Code	NAIG (*f*)	NAIG (*%*)	AIG (*f*)	AIG (*%*)
Type of Analogy	Direct Instruction	19	26.8	6	9.7
	Question/Discussion	4	5.6	27	43.5
	Story Format	38	53.5	23	37.1
	Gamified	10	14.1	6	9.7
Presentation of Analogy	Verbal	37	52.1	3	4.8
	Pictorial–Verbal	34	47.9	59	95.2
Semantic Distance	Close	54	76.1	6	9.7
	Close–Remote	17	23.9	56	90.3
Analogical Association	Structural	43	60.6	12	19.4
	Functional	5	7.0	3	4.8
	Structural–Functional	23	32.4	47	75.8
State of Abstraction	Concrete–Concrete	2	2.8	0	0.0
	Abstract–Abstract	3	4.2	4	6.5
	Concrete–Abstract	66	93.0	58	93.5
Wealth Level	Simple	39	54.9	6	9.7
	Enriched	27	38.0	24	38.7
	Extended	5	7.1	32	51.6
Limitations	Limitations in the use of analogy (Exist)	15	21.1	45	72.6
	Warning About Misleading Matches (Exist)	24	33.8	40	64.5
Source Domain	Anthropomorphic	5	7.0	11	17.7
	Environmental	28	39.5	33	53.2
	Anthropomorphic–Environmental	38	53.5	18	29.0

**Table 5:** Examples of the analogies designed by preservice science teachers.

Group	Analogy		
AIG	Topic: Life Cycle of Stars Analogy: The Fire Analogy for Stars 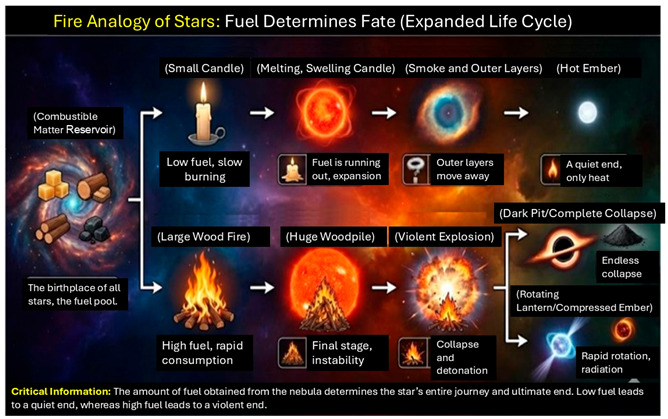 Questions		
	What are the differences between the life cycles shown in the two branches in the visual? Why does a small candle flame burn longer than a large wood fire? What does the difference between a fire that fades quietly and a fire that extinguishes explosively represent?		
	Structural Mapping Table: Let us match the elements in the table based on the analogy.		
	Fire Analogy (Source Domain)	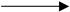	Star Formation/Characteristics of Stars
	Fuel Reservoir		Composed of gas and dust
	Small Candle Flame		The environment where stars are formed
	Large Wood Fire		The amount of matter determines the formation environment
	Growing and Burning Flame		Low-mass star
	Quietly Fading Fire and Smoke		Has a long lifespan
	Sudden Flare and Explosion		Consumes energy slowly
	Dense Glowing Ember Remaining		High-mass star
			Very bright and hot
			Has a short lifespan
			Red giant
			Red supergiant
			Planetary nebula
			White dwarf
			Supernova
			Neutron star
			Black hole
	**Note:** This analogy is intended solely to illustrate the relationship between stellar mass, energy output, and lifespan and should not be interpreted at the level of physical mechanisms. Stars do not obtain their fuel externally; their energy originates from hydrogen within their cores. Stars do not “burn” like fire; rather, energy is produced through nuclear fusion in their cores.		
	Source Domain: Fire—fuel—combustion process Target Domain: Star—mass—energy production—stellar life cycle Both visual and verbal elements were used in the analogy (pictorial–verbal presentation). The analogy was structured through a question–discussion format, encouraging learners to reflect on the relationships between the elements of the analogy. Since the analogy establishes a sequence of mass → energy consumption → lifespan → final stage, it enables close–remote semantic distance. The analogy includes both physical properties and process-based relationships, indicating a structural–functional analogical association. In terms of abstraction level, the analogy represents a concrete–abstract mapping. Due to its multi-stage process, alternative outcomes, causal relationships, and problem-based questions, the analogy can be categorized as an extended analogy. Furthermore, the limitations of the analogy and potential mismatches between the source and target domains are explicitly stated. As the analogy is based on a natural process rather than anthropomorphic, the source domain is classified as an environmental context.		
NAIG	Example Analogy: Library Analogy for the Solar System Topic: Solar System Analogy: Library		
	Nine students gathered in the university library to prepare a group assignment for their final project. The library was quite large and slightly cold, as it was winter. The heating system was operating. Before starting the assignment, each student planned to develop their own ideas and later compare them to complete the task collaboratively. The students sat at separate tables arranged in a row. The first table, closest to the heater, was occupied by Mehmet and Yılmaz. At the second table sat Kaya and Serdar. A row of bookshelves was located between the second and third tables. Buse sat at the third table, Sanem at the fourth, Yıldırım at the fifth, Fatih at the sixth, and Elif at the seventh table. Another row of bookshelves was located beyond the seventh table. Because Elif was particularly interested in history, she was distracted by the historical books on the nearby shelves and had difficulty focusing on the assignment. As the tables were positioned farther away from the heater, the temperature gradually decreased. For example, Mehmet and Yılmaz at the first table felt very warm because they were close to the heater, whereas Fatih and Elif at the sixth and seventh tables felt colder since they were farther away from the heater. Let us complete the table by considering the heater as the Sun and the students sitting at the tables as the planets. The bookshelves can also be compared to the asteroid belt.		
	Students Sitting at the Tables	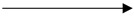	Planets (Target Domain—Solar System)
	Table 1—Mehmet & Yılmaz		Mercury, Venus
	Table 2—Kaya & Serdar		Earth, Mars
	Table 3—Buse		Jupiter
	Table 4—Sanem		Saturn
	Table 5—Yıldırım		Uranus
	Table 6—Fatih		Neptune
	Table 7—Elif		Pluto
	Source Domain: Seating arrangement and heat distribution in a library Target Domain: Planets in the Solar System In this analogy, verbal expressions were used as the primary form of representation. The analogy was structured in a story format, describing a scenario in which students sit at different tables in a library located at varying distances from a heater. Since the analogy explains the order of the planets and differences in temperature related to their distance from the Sun, it involves close semantic distance. The analogy is based on physical characteristics, indicating a structural analogical association. In terms of abstraction level, the analogy represents a concrete–abstract mapping, where a concrete everyday situation (seating arrangement in a library) is used to explain an abstract astronomical concept (the distribution of planets in the Solar System). Because the analogy focuses on two physical characteristics—distance and temperature—it can be classified as a simple analogy. Additionally, no limitations of the analogy or warnings about possible mismatches between the source and target domains are provided. Finally, since the analogy refers to a natural process rather than anthropomorphic characteristics, the source domain is categorized as an environmental context.		

**Table 6:** Chi-square analysis of the codes across analogy themes.

Theme	χ^2^	*df*	*p*	Cramer’s V
Type of Analogy	31.131	3	<.001	0.484
Presentation of Analogy	35.172	1	<.001	0.514
Semantic Distance	58.896	1	<.001	0.665
Analogical Association	25.710	2	<.001	0.440
State of Abstraction	2.059	2	>.05	0.124
Wealth Level	43.670	2	<.001	0.573
Limitations in the use of analogy (Exist)	35.389	1	<.001	0.516
Warning About Misleading Matches (Exist)	12.506	1	<.001	0.307
Source Domain	9.236	2	<.05	0.264

**Table 7:** Bonferroni-corrected post hoc pairwise comparisons of codes within analogy themes.

Theme	Code	NAIG	AIG	*p*
Type of Analogy	Direct Instruction	19a	6b	<.05
	Question/Discussion	4a	27b	<.05
	Story Format	38a	23b	<.05
	Gamified	10a	6a	>.05
Presentation of Analogy	Verbal	37a	3b	<.05
	Pictorial–Verbal	34a	59b	<.05
Semantic Distance	Close	54a	6b	<.05
	Close–Remote	17a	56b	<.05
Analogical Association	Structural	43a	12b	<.05
	Functional	5a	3a	>.05
	Structural–Functional	23a	47b	<.05
Wealth Level	Simple	39a	6b	<.05
	Enriched	27a	24a	>.05
	Extended	5a	32b	<.05
Limitations in the use of analogy	Exist	15a	45b	<.05
	Not Exist	56a	17b	<.05
Warning About Misleading Matches	Exist	24a	40b	<.05
	Not Exist	47a	22b	<.05
Source Domain	Anthropomorphic	5a	11a	>.05
	Environmental	28a	33a	>.05
	Anthropomorphic–Environmental	38a	18b	<.05

**Note:** Each subscript letter denotes a subset of group categories whose column proportions do not differ significantly from each other at the 0.05 level.

**Table 8:** Preservice science teachers’ views on AI-assisted analogy design.

Theme	Code	*f*
Purpose of AI Use	Idea generation	57
	Visual creation/enhancement	50
	Idea development/refinement	42
	Text editing/expression improvement	24
	Information search/research	23
	Story construction	16
	Prompt preparation	7
	Originality checking	5
Support Provided by AI	Visual support	55
	Time saving	54
	Creativity support	46
	Facilitating the process	38
	Diversity of ideas	35
	Creating a basic framework	8
AI Use Process	Selecting from AI-generated examples	47
	AI-assisted idea development	41
	Trial-and-error prompt writing	34
	Using multiple AI tools	25
Scientific Control When Using AI	Need to verify scientific accuracy	42
	Necessity of teacher supervision	34
	Comparing multiple sources	32
	Risk of generating incorrect information	29
Using AI and Writing Prompts	Learning how to write prompts	38
	Importance of clear instructions	29
	Training AI through iterative interaction	24
	Selecting appropriate AI tools	20
Preferred AI Tools	Gemini	51
	ChatGPT	45
	Canva	18
	Google Flow	6
	CapCut (AI-assisted editing)	1

## Data Availability

Data available on request due to restrictions.

## Appendix A

**Table A1:** Themes and codes definitions for preservice science teachers’ interview data on AI-supported analogy design.

Theme	Code	Code Definition
Purpose of AI use	Idea generation	Refers to using AI to generate initial analogy ideas, alternative source domains, or possible ways of explaining astronomy-related concepts through analogies.
	Visual creation/enhancement	Refers to using AI tools to create, improve, organize, or adapt visual materials, images, diagrams, or pictorial elements for analogy design.
	Idea development/refinement	Refers to using AI to improve, elaborate, reorganize, or make an initially developed analogy more understandable, coherent, or pedagogically appropriate.
	Text editing/expression improvement	Refers to using AI to revise wording, improve clarity, correct language, simplify explanations, or make written analogy descriptions more understandable for learners.
	Information search/research	Refers to using AI to obtain background information about astronomy concepts, analogy topics, scientific explanations, or possible instructional representations.
	Story construction	Refers to using AI to create or improve narrative-based analogies, storylines, scenarios, or contextualized explanations for astronomy concepts.
	Prompt preparation	Refers to using AI or deliberate planning to prepare prompts that guide AI tools toward producing relevant analogy ideas, visuals, or explanations.
	Originality checking	Refers to using AI to check whether the analogy idea is original, different from common examples, or sufficiently creative for instructional use.
Support provided by AI	Visual support	Refers to AI’s contribution to producing, improving, or selecting visual representations that support the analogy design process.
	Time saving	Refers to participants’ perception that AI accelerated the analogy design process by quickly generating ideas, visuals, explanations, or alternative examples.
	Creativity support	Refers to AI’s role in helping participants think of more creative, unusual, diverse, or pedagogically engaging analogy ideas.
	Facilitating the process	Refers to AI’s general role in making the analogy design process easier, more manageable, or more structured for preservice science teachers.
	Diversity of ideas	Refers to AI’s role in offering multiple alternatives, different perspectives, or varied source domains that participants could compare and select from.
	Creating a basic framework	Refers to using AI to generate an initial structure, outline, or draft framework for the analogy before further revision by the participant.
AI use process	Selecting from AI-generated examples	Refers to participants reviewing multiple AI-generated analogy suggestions and choosing the one they considered most appropriate.
	AI-assisted idea development	Refers to a process in which participants developed their own initial ideas further with the help of AI-generated suggestions, explanations, or revisions.
	Trial-and-error prompt writing	Refers to repeatedly revising, reformulating, or testing prompts to obtain clearer, more accurate, or more useful AI outputs.
	Using multiple AI tools	Refers to using more than one AI tool for different purposes, such as idea generation, visual creation, design, editing, or simplification.
Scientific control when using AI	Need to verify scientific accuracy	Refers to checking whether AI-generated explanations, analogies, visuals, or source–target mappings are scientifically accurate and consistent with astronomy content.
	Necessity of teacher supervision	Refers to the need for teacher or instructor guidance to evaluate AI-generated outputs, prevent misconceptions, and ensure pedagogical appropriateness.
	Comparing multiple sources	Refers to checking AI-generated information against textbooks, course materials, reliable websites, or other AI tools to confirm accuracy.
	Risk of generating incorrect information	Refers to participants’ awareness that AI may produce scientifically inaccurate, misleading, incomplete, or inappropriate information.
Using AI and writing prompts	Learning how to write prompts	Refers to participants’ development of awareness and skill in writing prompts during their interaction with AI tools.
	Importance of clear instructions	Refers to participants’ realization that clear, specific, and well-structured prompts lead to more relevant and useful AI outputs.
	Training AI through iterative interaction	Refers to guiding AI step by step by revising prompts, providing additional instructions, correcting outputs, or asking follow-up questions.
	Selecting appropriate AI tools	Refers to participants’ awareness that different AI tools may be more suitable for different purposes, such as text generation, visual creation, or design.
Preferred AI tools	Gemini	Refers to participants’ reported use of Gemini during analogy design, particularly for idea generation, visual creation, or explanation development.
	ChatGPT	Refers to participants’ reported use of ChatGPT during analogy design, particularly for prompt preparation, idea generation, simplification, or text revision.
	Canva	Refers to participants’ reported use of Canva or AI-assisted Canva features for visual design, layout preparation, or presentation of analogy materials.
	Google Flow	Refers to participants’ reported use of Google Flow for AI-supported visual, media, or design-related purposes during analogy preparation.
	CapCut (AI-assisted editing)	Refers to participants’ reported use of CapCut’s AI-assisted editing features for preparing or editing visual/video-based analogy materials.
